# Uniaxial Compression Behavior of Cement Mortar and Its Damage-Constitutive Model Based on Energy Theory

**DOI:** 10.3390/ma12081309

**Published:** 2019-04-22

**Authors:** Yunliang Tan, Qingheng Gu, Jianguo Ning, Xuesheng Liu, Zhichuang Jia, Dongmei Huang

**Affiliations:** 1 State Key Laboratory of Mining Disaster Prevention and Control Co-founded by Shandong Province and the Ministry of Science and Technology, Shandong University of Science and Technology, Qingdao 266590, China; yunliangtan@163.com (Y.T.); njglxh@126.com (J.N.); xuesheng1134@163.com (X.L.); kmcandy@126.com (D.H.); 2 School of Mining and Safety Engineering, Shandong University of Science and Technology, Qingdao 266590, China; jiazc@yuhong.com.cn

**Keywords:** cement mortar, mechanical properties, energy dissipation, constitutive model

## Abstract

The mechanical properties of mortar materials in construction are influenced both by their own proportions and external loads. The trend of the stress–strain curve in cracks compaction stage has great influence on the relationship between the strength and deformation of cement mortar. Uniaxial compression tests of mortar specimens with different cement–sand ratios and loading rates were carried out, and the stored and dissipated energies were calculated. Results indicated that the elastic modulus and strength of mortar specimens increase with the cement–sand ratio and loading rate. The energy dissipation shows good consistency with the damage evolution. When the loading rate is less than 1.0 mm/min, most of the constitutive energy at the peak point is stored in the specimen and it increase with cement–sand ratio. A simple representation method of axial stress in cracks compaction stage was proposed and an energy-based damage constitutive model—which can describe well the whole process of cement mortar under uniaxial compression—was developed and verified.

## 1. Introduction

Mortar materials are widely used in construction and mining engineering. Their use has effectively improved our living conditions and protected the ecological environment by filling the mined-out area [1,2,3,4,5]. In recent years, with the development of science and technology and the improvement of functionality and safety requirements for construction projects, more and more new materials and complex structures emerge, such as gravity offshore platform, high arch dam, long-span bridge, etc., and engineering design and evaluation methods have been developed.

Generally, no matter what kind of mortar material and engineering structure, its mechanical properties are determined by both internal factors (material composition, internal structure, etc.) and external factors (surrounding environment, stress characteristics, etc.) [6,7,8,9,10,11,12]. For cement mortar made of same materials, the mechanical properties are different when the material mixture ratio is different, and the mechanical properties of the specimens are also different when the loading path is different. Therefore, we should consider both internal and external factors when we study the mechanical properties of a mortar material, so as to guide engineering design with higher efficiency.

As for the influence of internal factors on the properties of mortar materials, researchers usually study the influence of material components on the properties of mortar by controlling variable method, such as S. Firoozi et al. [11] and J.K. Zhou et al. [13] studied the influence of water–cement ratio on the properties of mortar. As for the influence of external factors on the performance of mortar materials, researchers mostly focus on improving material properties to resist external heavy loads or chemical erosion. For example, some researchers maintain the long-term stability of mortar materials by adding some anti-corrosion components to the mortar [14,15,16,17]. However, studies have shown that the deformation response of solid materials is not only related to the load level, but also to the loading rate, which is often overlooked in the study of mechanical properties of mortar.

The relationship between the strength and deformation of mortar material is the foundation for design and assessment of related construction projects. Many researchers have made contributions to the understanding of the strength and deformability of mortar or rock-like materials, and many damage constitutive models of materials are established from the point of view of degradation of elastic modulus, energy dissipation, etc. [18,19,20,21,22,23]. Moreover, some researchers established a variety of damage models based on statistical mathematics theory or with the aid of acoustic emission monitoring technology [24,25,26]. However, these constitutive models are based on the premise that the initial damage of the material is 0 before the compression test. It is well known that there are abundant primary defects in mortar materials or rock-like materials. Compared with intact material samples, we can conclude that mortar or rock-like material samples have initial damage. Based on this viewpoint, Yang et al. [27] established a damage constitutive model of coal considering initial damage under triaxial compression, which describes well the stress–strain relationship of coal under triaxial compression. However, the compaction effect of cracks has a significant influence on the trend of the stress–strain curve. The concave phenomenon of stress–strain curve caused by cracks compression is neglected in establishing the constitutive model [27], and especially for mortar specimens with abundant defects under uniaxial compression. Therefore, the aim of this research is to obtain the mechanical properties of cement mortar and a constitutive model which can describe appropriately the relationship between the strength and deformation, by properly describing the non-linear stress–strain relationship in the compaction stage. The most commonly used cement mortar for mine filling (cement and sand composition) is taken as the research object. The energy dissipation characteristics of cement mortar with different cement–sand ratio under uniaxial compression are studies, and a constitutive model with modified damage was developed.

## 2. Materials and Methods

### 2.1. Materials and Mixing Ratio

Ordinary Portland cement (OPC) 42.5R grade, ordinary river sand, and potable water were used for preparing test specimens. The relative density of OPC is 3.1 g/cm^3^, and the chemical composition is shown in Table 1. The physical and mechanical properties of cement were tested according to GB/T 1346-2011 [28] and GB/T 17671-1999 [29]. The test results are shown in Table 2. 

**Figure 1:**
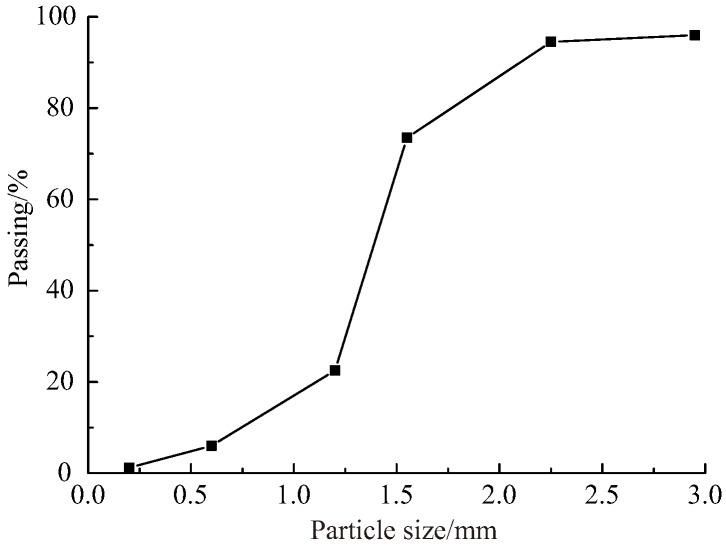
Particle size distribution of the river sand.

The mineral composition of river sand is mainly quartz, followed by feldspar and clay. The physical properties of the sand are shown in Table 3. The particle size distribution of the natural river sand used in this program is shown in Figure 1. About seventy percent of the river sand is 1.2~2.2 mm in diameter. The potable water used in the experiment was taken from the laboratory. To study the influence of internal factors on the properties of cement mortar, three different proportions of cement mortar were designed to study the mechanical property with different cement–sand ratios, that is cement–sand ratios of 1:2 (Group A), 1:1.5 (Group B), and 1:1 (Group C), and the water–cement ratio of the three groups of samples remains the same, that is 1:2.3. Each specimen with a specific mix proportion is shown in Table 4.

### 2.2. Sample Preparation

**Figure 2:**
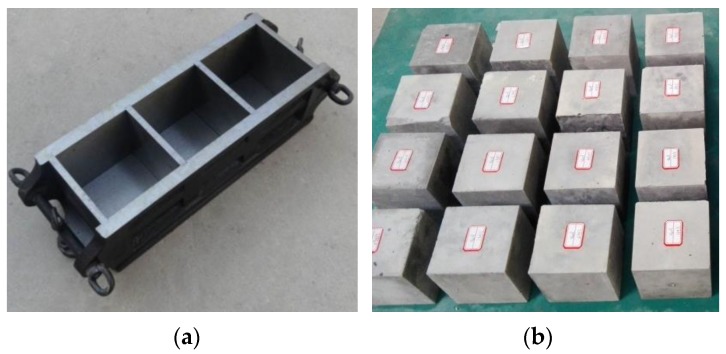
Manufacture of mortar samples: (**a**) Standard metallic cube mold; (**b**) Mortar specimens after curing 28 d.

The cement and river sand were mixed in the designed proportion and stirred until the mixture was even. Then the designed amount of water was added and the cement mortar slurry mixed according to GB/T 17671-1999 [29]. The slurry with uniform mixing had good workability, and no mixing agglomeration and segregation appeared. Cube mortar specimens with edge length of 70.7 mm were prepared by using the standard metallic cube molds, as shown in Figure 2a. Three batches (Group A, B, and C) of mortar cubes for compression tests were prepared in accordance with JGJ/T 70-2009 [30]. After 24 h, all specimens were removed from the mold and put in curing tank at a constant temperature (20 ± 1 °C) and humidity (not less than 90%) up till the age of 28 days. Figure 2b shows part of the samples after curing 28 d.

### 2.3. Test Methods

**Figure 3:**
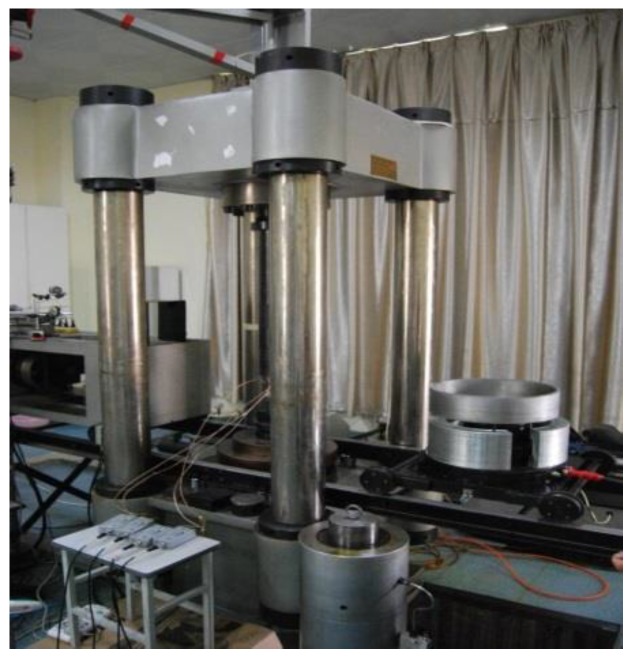
RLJW-2000-type test system.

**Figure 4:**
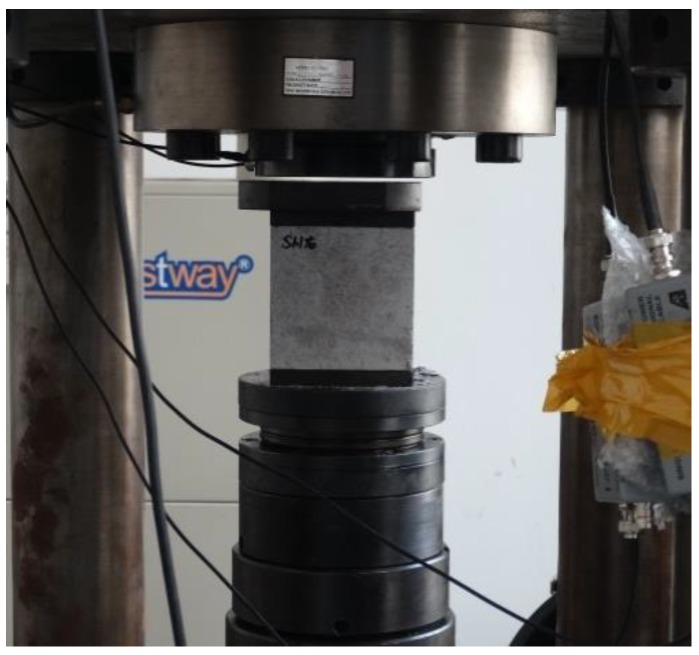
Sample placement and compression.

After the prescribed curing periods, the unconfined compression strength (UCS) of the three groups of specimens was measured by RLJW-2000 servo-controlled testing machine (Chaoyang test instrument Co., Ltd., Changchun city, China), as shown in Figure 3. The load system was controlled through a displacement control mode. To study the influence of external factors on the performance of cement mortar, the tests were performed at a constant loading speed of 0.1 mm/min 0.5 mm/min 1.0 mm/min, respectively. The axial deformation of the specimen is obtained by monitoring the displacement of the test-bed for placing samples (shown in Figure 4), and the axial strain of the specimen is obtained by comparing the axial displacement with the height of the specimen. To ensure repeatability of test, 3 mortar specimens were tested repeatedly in the same stress state in each case, and the average strength of the three specimens regarded as the UCS of the cement mortar under these conditions. The experimental operation process was based on GB/T 50081-2002 [31].

## 3. Results and Discussion

### 3.1. Uniaxial Compression Behavior

The strength and deformation characteristics of mortar specimens with different cement–sand ratios and loading rates are listed in Table 4. The unconfined compression strengths (UCS) and elastic modulus (*E*) of cement mortar with a same loading rate increased with the cement–sand ratio. Taking the mortar samples with a loading rate of 0.5 mm/min as an example, the UCS increased by 25.58% and 51.32%, and the *E* increased by 7.97% and 44.53%, respectively, as the cement–sand ratio increased from 1:2 to 1:1.5 and from 1:2 to 1:1. This trend repeated for all three groups of test specimens, which showed that the compressive mechanical properties of the specimens were improved with an increase in the cement–sand ratio.

Similarly, the UCS and *E* of mortar samples with a same cement–sand ratio increased with the loading rate. For mortar samples with cement–sand ratio of 1:1.5, compared with mortar samples with a loading rate of 0.1 mm/min, the UCS of mortar samples with a loading rate of 0.5 mm/min and a loading rate of 1.0 mm/min increased by 7.69% and 14.79%, respectively. This shows that the influence of the cement–sand ratio on the mechanical properties of cement mortar is more obvious than that of the loading rate on the mechanical properties of cement mortar.

### 3.2. Energy Accumulation and Dissipation Characteristics

From the viewpoint of energy, the deformation and damage of rock-like materials is a process of energy input, elastic energy accumulation, energy dissipation and energy release [32]. According to the law of conservation of energy, part of the work done by an external force is stored in the material as elastic energy, and the other part is dissipated. The relationship between the constitutive energy, elastic energy and dissipative energy, and their calculation formula are as follows [33].

(1) $$U=U^{e}+U^{d}$$

(2) $$U=\int \sigma_{1}d\varepsilon_{1}=\sum_{i=1}^{n}\frac{1}{2}\left(\sigma_{1i}+\sigma_{1i-1}\right)\left(\varepsilon_{1i}-\varepsilon_{1i-1}\right)$$

(3) $$U^{e}=\frac{\sigma_{1}^{2}}{2E_{i}}$$

where *U* is the constitutive energy, *U^e^* is the elastic strain energy, and *U^d^* is the dissipated energy; *σ*_1_ and *ε*_1_ are the axial stress and strain; *σ*_1i_ and *ε*_1i_ are the stress and strain values at each point of the stress–strain curve. *E_i_* is the unloading elastic modulus, for the convenience of calculation, the effective elastic modulus *E* can be used instead. 

**Figure 5:**
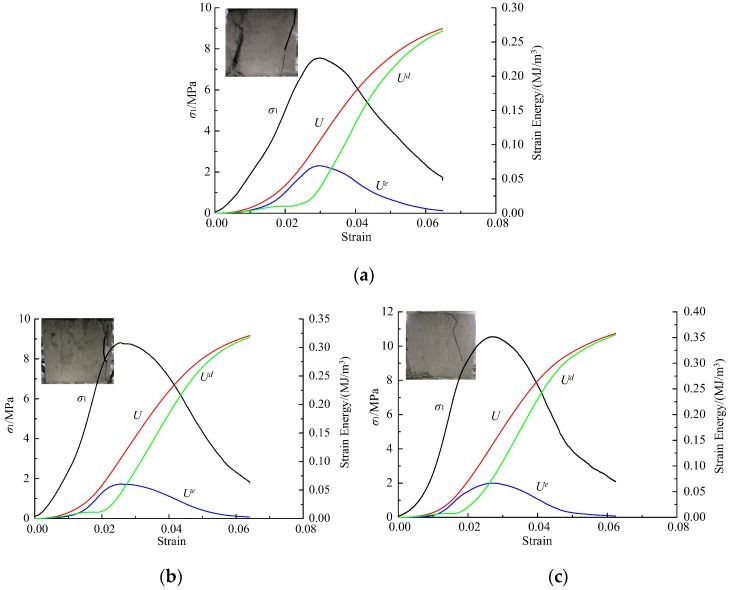
Stress and energy with strain for cement mortar samples: (**a**) A-0.1–1; (**b**) A-0.5–2; (**c**) A-1.0–1.

**Figure 6:**
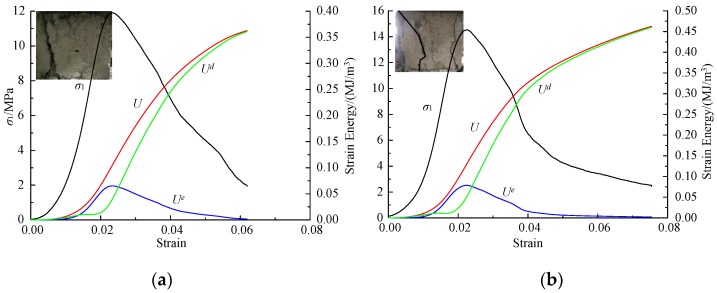
Stress and energy with strain for cement mortar samples: (**a**) B-1.0–3; (**b**) C-1.0–1.

The stress–strain curves and evolution trend of energy curves in mortar specimens with different cement–sand under different loading rates is basically the same. Since nine cases of experiments were carried out and three samples were tested for each case, the results of the experiments mentioned above are numerous. Five samples representing three different cement–sand ratios and loading rates were selected, as shown in Figure 5 and Figure 6. It should be noted that A-0.1–1 represents the sample No. 1 with loading rate of 0.1 mm/min in Group A, and the same as other samples. At the initial stage of loading, the stress–strain curve is concave and the input energy is mainly used for pore and crack compression. When the pores and cracks are compressed and closed, the elastic energy accumulated in the specimen increases rapidly, and the dissipated energy increases little. After the elastic stage, the elastic energy accumulated in the specimen gradually releases, and the dissipated energy increases rapidly. Therefore, we can obtain the damage evolution of the specimen through the energy change during the loading process. The difference is that the constitutive energy of mortar samples with the same cement–sand ratio increases with the loading rate, and that of mortar samples increases with the cement–sand ratio under the same loading rate. A special phenomenon is that the crack-compaction effect of the stress–strain curve of mortar specimens is very obvious when the loading rate is greater than 0.5 mm/min. Therefore, in describing the stress–strain relationship of mortar specimens, the influence of crack compaction on the curve trend cannot be ignored.

Table 1 also lists the energy storage and dissipation of mortar specimens under different loading rates. When the loading rate is less than 1.0 mm/min, the difference between the constitutive energy and elastic energy of specimens with the same cement–sand ratio at the peak point is very small. For example, when the cement–sand ratio is 1:2 and the loading rate is increased from 0.1 mm/min to 0.5 mm/min, the constitutive energy of specimens at the peak point remains about 0.110 MJ/m^3^, and the elastic energy remains about 0.065 MJ/m^3^. The constitutive energy and elastic energy of mortar specimens with a loading rate less than 1.0 mm/min at the peak point increase with the cement–sand ratio.

### 3.3. Damage Constitutive Model for Cement Mortar 

#### 3.3.1. Damage Evolution Equation

Damage mechanics is a developing science. Many studies show that the damage of solid material can be expressed by energy dissipation. For example, Jin et al. [34] defined and calculated the damage variable based on energy dissipation for rock subjected according to monotonic or cyclic loading. The usual damage variable (D) can be expressed as follows:

(4) $$D_{P}=\frac{U_{P}^{d}}{U_{P}}=\frac{U_{P}-U_{P}^{e}}{U_{P}}$$

where *U_P_*, $U_{p}^{d}$, and $U_{p}^{e}$ are the constitutive energy, dissipated energy, and elastic energy at point P, respectively.

**Figure 7:**
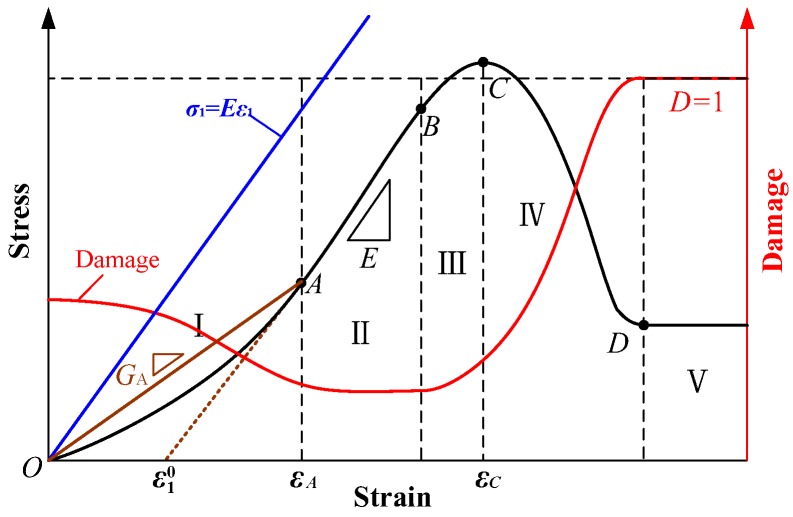
Stress–strain-damage curves of rock-like materials during the compression process.

Furthermore, many experiment results have proved that the stress–strain curve of rock-like materials during compression can be divided into five stages [18,35,36]: a crack-compaction stage (I), a quasilinear elastic stage (II), a yield stage (III), a failure stage (IV), and a residual strength stage (V), as shown in Figure 7. Points A to D represent cracks compression closure point, yield point, peak point and residual point of materials, respectively.

**Figure 8:**
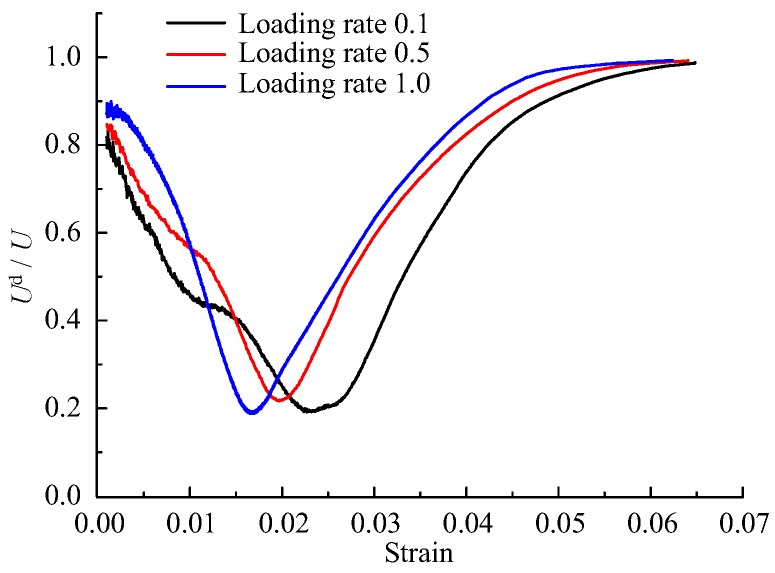
Energy dissipation ratio of specimens with cement/sand = 1:2.

According to the calculation method of constitutive energy and dissipated energy in Section 3.2, we can get the evolution of energy dissipation ratio (ratio of dissipated energy to constitutive energy) of mortar specimen under different loading rates. Figure 8 is the calculation result of energy dissipation ratio of mortar specimens with cement–sand ratio of 1:2. In the cracks compaction stage, most of the input energy is used for the compression of cracks. After the crack-compaction stage, most of the input energy is stored in the specimen in the form of elastic energy. However, there is still a certain proportion of energy dissipation at this time, which indicates that the whole compression process is accompanied by damage. Even in the elastic stage with the least damage, the ratio of dissipated energy will be nearly 20%.

The energy dissipation at the crack-compaction stage is not caused by material damage. On the contrary, it is caused precisely because part of the energy input is used for the compaction of cracks, which improves the load-bearing capacity of the specimen. Therefore, the expression of damage variable in Equation (4) cannot really reflect the change of rock internal structure at the compaction stage. Based on this, some scholars define primary fractures as initial damage. Additionally, the damage evolution is shown in Figure 7. The damage variable at point P on stress–strain curve expressed using energy dissipation $U_{P}^{d}$ is as follows [27].

(5) $$D_{P}=1-\exp\left\{-\alpha {\left[{\left(\frac{U_{P}^{d}-U_{0}^{d}}{U_{0}}\right)}^{2}\right]}^{\beta }\right\}$$

where *U*_0_ = 1 mJ mm^−3^ is the unit strain energy after dimensionless treatment; *α* and *β* are parameters related to material properties; $U_{0}^{d}$ is the dissipated energy corresponding to the initial damage. 

The rock will damage under external load, accompanied by energy dissipation. The dissipation energy $U_{p}^{d}$ in Equation (5) can be shown as follows:

(6) $$U_{P}^{d}=\frac{J_{2}}{2G}=\frac{\left[{\left(\sigma_{1}-\sigma_{2}\right)}^{2}+{\left(\sigma_{2}-\sigma_{3}\right)}^{2}+{\left(\sigma_{3}-\sigma_{1}\right)}^{2}\right]}{12G}$$

where *G* is the shear modulus, and *J*_2_ is the second invariant of the stress deviator tensor.

In uniaxial compression testing, *σ*_2_ = *σ*_3_ = 0. Therefore, substituting it into Equation (7), we have

(7) $$U_{P}^{d}=\frac{\sigma_{1}^{2}}{6G}=\frac{\left(1+\upsilon \right)}{3E}\sigma_{1}^{2}$$

The slope of stress–strain curve at the stage of cracks compaction is obviously smaller than that of elastic modulus *E* due to the effect of cracks compaction at the initial stage of loading. We cannot use *Eε* to represent *σ*_1_, as shown in Figure 7. Here we present a simple representation of *σ*_1_, it can be expressed as:

(8) $$\sigma_{1}=\left\{ \begin{array}{ll} G_{A}\varepsilon & \varepsilon <\varepsilon_{A}\\ E\varepsilon -\left(E-G_{A}\right)\varepsilon_{A} & \varepsilon \geq \varepsilon_{A} \end{array}\right.$$

(9) $$G_{A}=\frac{\varepsilon_{A}-\varepsilon_{1}^{0}}{\varepsilon_{A}}E$$

where *G_A_* is the slope of line OA. *ε_A_* is the total strain at the crack closure point A. $\varepsilon_{1}^{0}$ is the plastic strain at point A.

Therefore, substituting Equations (8) and (9) into Equation (7), we have

(10) $$U_{P}^{d}=\left\{ \begin{array}{ll} \frac{E\left(1+\upsilon \right)}{3}{\left(\frac{\varepsilon_{A}-\varepsilon_{1}^{0}}{\varepsilon_{A}}\right)}^{2}\varepsilon^{2} & \varepsilon <\varepsilon_{A}\\ \frac{E\left(1+\upsilon \right)}{3}{\left(\varepsilon -\varepsilon_{1}^{0}\right)}^{2} & \varepsilon \geq \varepsilon_{A} \end{array}\right.$$

#### 3.3.2. Damage Constitutive Model and Verification

Through the obvious compaction stage of stress–strain curves, it can be seen that there are a lot of primary defects in the mortar sample. The deformation of a specimen during compression includes the elastic deformation of the intact material and the plastic deformation of cracks. Therefore, the damage constitutive equation of mortar material can be expressed as [27]

(11) $$\sigma_{ij}=\sigma_{ij}^{e}(1-D)+\sigma_{ij}^{cr}D$$

(12) $$\sigma_{ij}^{e}=C_{ijkl}(\varepsilon_{kl}-\varepsilon_{kl}^{0})$$

where $\sigma_{ij}^{e}$ and $\sigma_{ij}^{cr}$ are the axial stresses applied on the intact part and defective part, respectively. *C_ijkl_* and *ε_kl_* are the elastic coefficient and elastic strain of the material, respectively. $\varepsilon_{kl}^{0}$ is the strain of the defective part.

Mohr–Coulomb strength criterion has simple parameter form and it is applicable for rock-like material [37,38]. It can be used to describe the mechanical behavior of cement mortar in this research. The expression of Mohr–Coulomb strength criterion under uniaxial compression can be shown as follows:

(13) $$f\left(\sigma_{1},D\right)=\sigma_{1}-C=\sigma_{1}-\frac{2c\left(D\right)\cos\varphi \left(D\right)}{1-\sin\varphi \left(D\right)}$$

where *φ*(*D*) is the internal friction angle and *c*(*D*) is the internal cohesion of cement mortar. Additionally, they can all be expressed as functions of damage variable D. Combining Equations (5) and (10)–(13), the nonlinear short-term damage model can be produced.

**Figure 9:**
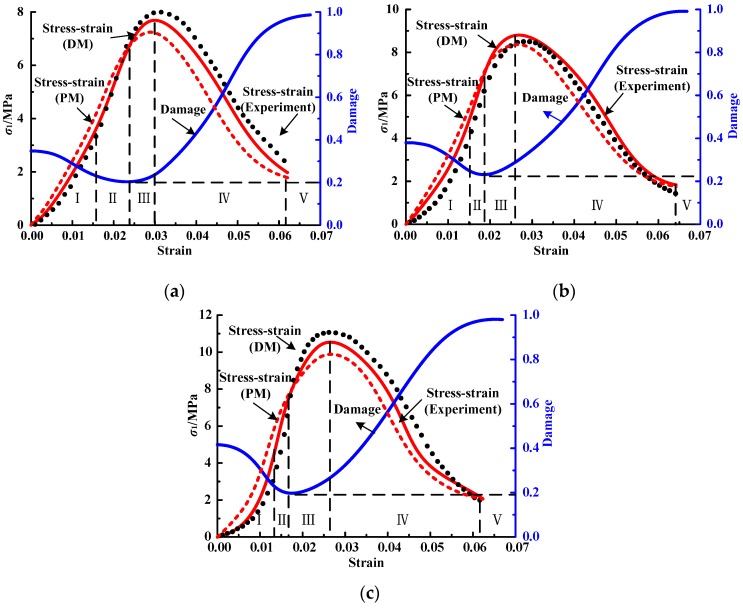
Comparison between experimental and theoretical curves of cement mortar: (**a**) A-0.1–2; (**b**) A-0.5–3; (**c**) A-1.0–2.

**Figure 10:**
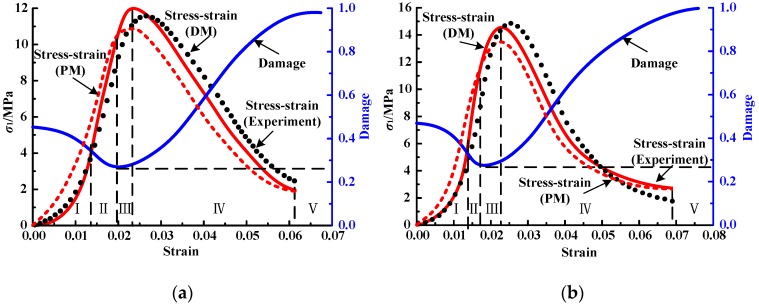
Comparison between experimental and theoretical curves of cement mortar: (**a**) B-1.0–2; (**b**) C-1.0–3.

Based on the stress and strain data of mortar specimens as used in Section 3.2, parameters *α*, *β* and $U_{0}^{d}$ of the damage constitutive model can be fitted by Equations (5), (7) and (11)–(13), as shown in Table 5. Then the stress and strain data of the other five specimens in the same case as those mentioned in Section 3.2 are taken, and the theoretical curves can be obtained, as shown in Figure 9 and Figure 10. To show the advantages of the developed model (DM) by comparison, the theoretical curve obtained by the previous model (PM) without considering the effect of cracks compaction [27] is also plotted in Figure 9 and Figure 10. It can be seen that the stress–strain curves described by PM are generally higher than experimental result before yielding point and lower than the test value after yielding point. While the stress–strain curve described by DM is basically consistent with the experimental curve, especially in the crack-compaction stage, the theoretical curve is in good agreement with the experimental curve, which also determines that the trend deviation of the stress–strain curve after crack compression closure is small.

**Figure 11:**
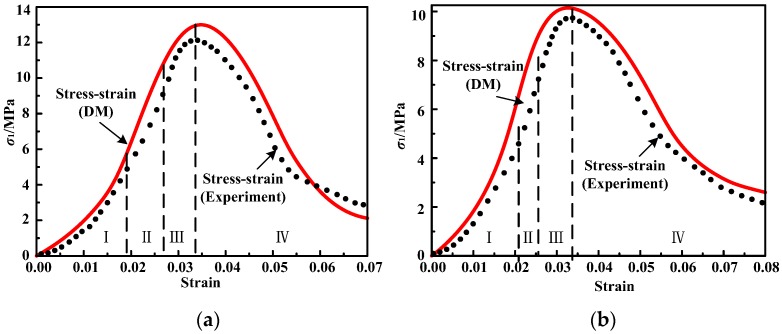
Comparison between experimental and theoretical curves of cement mortar: (**a**) cement–sand ratio of 1:1 and loading rate of 0.1 mm/min; (**b**) cement–sand ratio of 1:2 and loading rate of 1.0 mm/min.

To further observe the applicability of the developed model, a comparison between the proposed model and experimental data of two kinds of cement mortar in References [6,39] was made. The cement–sand ratio, elastic modulus *E*, total strain *ε_A_* at the crack closure point, plastic strain $\varepsilon_{1}^{0}$ at the crack closure point, residual strength *σ_cr_*, and the loading rate are 1:1 and 1:2, 0.925 GPa and 0.837 GPa, 0.019 and 0.021, 0.009 and 0.008, 2.86 MPa and 2.08 MPa, 0.1 mm/min and 1.0 mm/min, respectively. The parameters in the damage variable can be found from Table 5 according to the cement–sand ratio and loading rate. The comparison between the theoretical curve obtained by the developed model and the experimental data is shown in Figure 11. The comparison results show that the damage constitutive model developed in this paper can also describe the stress–strain relationship of the selected cement mortar specimens under uniaxial compression well.

## 4. Conclusions

Uniaxial compression behavior and energy accumulation and dissipation characteristics of cement mortar with different cement–sand ratio and loading rates were studied in this paper. A damage constitutive model considering the crack-compaction effect and initial damage was reported and validated by comparing the experimental and theoretical results of previous model and developed model. According to the results obtained, some conclusions can be made.

(1) The unconfined compression strengths and elastic modulus of cement mortar increased with the cement–sand ratio and loading rate. At the initial stage of loading, the stress–strain curve of the specimen is concave due to the compaction of cracks and pores, which will affect the trend of the curve after the compaction stage. The constitutive energy of the specimen mainly contributes to the compression and closure of cracks and pores.

(2) The elastic energy accumulation increases rapidly and more than 20% of the constitutive energy is still dissipated in elastic stage. Most of the constitutive energy at the peak point is stored in the specimen, and the constitutive energy and elastic energy at the peak point increase with the cement–sand ratio when the loading rate is less than 1.0 mm/min. This indicates that the influence of the cement–sand ratio on the mechanical properties of cement mortar is more obvious than that of the loading rate.

(3) A sample representation method of axial stress was proposed, and an energy-based constitutive model considering initial damage for cement mortar was developed. It can describe appropriately the stress–strain relationship of cement mortar with different cement–sand ratio.

(4) We should pay more attention to the characteristics of materials themselves than the external environment in engineering design. The damage constitutive model proposed in this paper is suitable for materials with abundant primary defects and obvious compaction stage. However, for materials that the compaction stage is not obvious, the stress–strain relationship can be described in an acceptable error without simply expressing its axial stress. In addition, crack-compaction effect will be limited under confining pressure, and the significance of the existence of the model needs to be further explored.

## Figures and Tables

**Table 1:** Chemical composition of OPC.

Chemical Composition	S_i_O_2_	Fe_2_O_3_	Al_2_O_3_	CaO	MgO	SO_3_
Percentage	22.4	3.15	5.6	59.58	2.58	2.42

**Table 2:** Physical properties of OPC.

S.No.	Physical Requirements	Test Results	GB/T 1346-2011 or 17671-1999 Requirement
1	Fineness modulus	4.4	10 Max.
2	Water requirement of normal consistency	26.8	30 Max.
3	Setting time (Minutes)		
	Initial	195	45 Min.
	Final	260	600 Max.
4	Soundness	1.5	5.00 Max.
5	Compressive strength (Mpa)		
	3 days ± 1 h	27.5	17.00 Min.
	28 days ± 4 h	49.2	42.50 Min.

**Table 3:** Physical properties of the river sand.

Property	Specific Gravity	Bulk Density	Fineness Modulus	Silt Content	Porosity
Values	2.46	1610 kg/m^3^	2.5	2.4%	38.2%

**Table 4:** Mechanical properties, energy storage and dissipation of mortar specimens.

Group	Cement/Sand	Loading Rate (mm/min)/(mm/min)	*E* (Gpa)	*σ_E_*	UCS (MPa)	*σ_UCS_*	*U*_C_ (MJ/m^3^)	$U_{C}^{e}$ (MJ/m^3^)
A	1:2	0.1	0.411	0.032	7.550	0.426	0.109	0.069
		0.5	0.640	0.044	8.805	0.458	0.112	0.060
		1.0	0.837	0.042	10.528	0.843	0.140	0.068
B	1:1.5	0.1	0.608	0.026	10.373	0.726	0.132	0.086
		0.5	0.691	0.033	11.057	0.551	0.137	0.088
		1.0	1.087	0.049	11.907	0.963	0.106	0.065
C	1:1	0.1	0.696	0.035	12.168	0.855	0.161	0.106
		0.5	0.925	0.048	13.324	0.560	0.169	0.096
		1.0	1.339	0.067	14.531	0.922	0.131	0.079

*E* = *E*_1_ + *E*_2_ + *E*_3_, *UCS* = *UCS*_1_ + *UCS*_2_ + *UCS*_3_, *E*_1_, *E*_2_ and *E*_3_, *UCS*_1_, *UCS*_2_ and *UCS*_3_ are elastic modulus and uniaxial compressive strength of three specimens in the same case, respectively. *σ_E_* and *σ_UCS_* are standard deviations of *E* and *UCS* values in each case. *U_C_* and $U_{C}^{e}$ are the constitutive energy and elastic strain energy at peak point C, respectively.

**Table 5:** Parameters of damage constitutive model of cement mortar specimens.

Group	Cement/Sand	Loading Rate (mm/min)	*ε_A_*	$\varepsilon_{1}^{0}$	*σ_cr_* (MPa)	*α*	*β*	$U_{0}^{d}$ (MJ/m^3^)
A	1:2	0.1	0.0166	0.0077	1.71	8.66 × 10^−6^	1.17	1.34
		0.5	0.0143	0.0070	1.82	1.24 × 10^−8^	1.59	0.87
		1.0	0.0118	0.0071	2.08	1.16 × 10^−6^	1.04	0.75
B	1:1.5	0.1	0.0225	0.0136	2.03	3.18 × 10^−12^	1.22	3.54
		0.5	0.0230	0.0140	4.18	5.62 × 10^−8^	1.58	1.59
		1.0	0.0145	0.0097	1.93	1.36 × 10^−8^	1.20	0.63
C	1:1	0.1	0.0242	0.0104	2.78	6.84 × 10^−22^	3.32	4.99
		0.5	0.0228	0.0146	2.06	1.32 × 10^−7^	1.16	2.82
		1.0	0.0131	0.0090	2.51	1.56 × 10^−8^	2.34	0.74

*ε_A_* and $\varepsilon_{1}^{0}$ are the total strain and plastic strain at the crack closure point A, respectively. *σ_cr_* is the residual strength. *α*, and *β* are parameters related to material properties; $U_{0}^{d}$ are is the dissipated energy corresponding to the initial damage.

## References

[1] Arif M., Gupta V., Choudhary H., Kumar S. (2018). Performance evaluation of cement concrete containing sandstone slurry. Constr. Build. Mater..

[2] Lin K., Totoev Y.Z., Liu H.J., Wei C.L. (2015). Experimental Characteristics of Dry Stack Masonry under Compression and Shear Loading. Materials.

[3] Folagbade S.O. (2017). Early-age performance of cement combination concrete. Civ. Eng. Dim..

[4] Tan Y.L., Yu F.H., Ning J.G., Zhao T.B. (2015). Design and construction of entry retaining wall along a gob side under hard roof stratum. Int. J. Rock Mech. Min..

[5] Torres S.M., Sharp J.H., Swamy R.N., Lynsdale C.J., Huntley S.A. (2003). Long term durability of Portland-limestone cement mortars exposed to magnesium sulfate attack. Cem. Concr. Comp..

[6] Liu X.S., Gu Q.H., Tan Y.L., Ning J.G., Jia Z.C. (2019). Mechanical Characteristics and Failure Prediction of Cement Mortar with a Sandwich Structure. Minerals.

[7] Dong B.Q., Qiu Q.W., Xiang J.Q., Huang C.J., Xing F., Han N.X. (2014). Study on the Carbonation Behavior of Cement Mortar by Electrochemical Impedance Spectroscopy. Materials.

[8] Barnat-Hunek D., Widomski M.K., Szafraniec M., Łagód G. (2018). Impact of Different Binders on the Roughness, Adhesion Strength, and Other Properties of Mortars with Expanded Cork. Materials.

[9] Ma Q., Tan Y.L., Zhao Z.H., Xu Q., Ding K., Wang J. (2018). Roadside support schemes numerical simulation and field monitoring of gob-side entry retaining in soft floor and hard roof. Arab. J. Geosci..

[10] Ilja F., Bernhard P., Erhardt L., Christina T., Elodie B., Fabienne B. (2014). Compressive strength of cement paste as a function of loading rate: Experiments and engineering mechanics analysis. Cem. Concr. Res..

[11] Firoozi S., Dehestani M.B., Neya N. (2018). Effect of water to cement ratio on the mode III fracture energy of self-compacting concrete. Mater Struct..

[12] Kong B., Wang E.Y., Li Z.H. (2018). The effect of high temperature environment on rock properties—An example of electromagnetic radiation characterization. Environ. Sci. Pollut. Res..

[13] Zhou J.K., Ge L.M. (2015). Effect of strain rate and water-to-cement ratio on compressive mechanical behavior of cement mortar. J. Cent. South Univ..

[14] Heidari A., Hashempour M., Javdanian H., Karimian M. (2018). Investigation of mechanical properties of mortar with mixed recycled aggregate. Asian J. Civ. Eng..

[15] Wahab M.A., Latif I.A., Kohail M., Almasry A. (2017). The use of Wollastonite to enhance the mechanical properties of mortar mixes. Constr. Build. Mater..

[16] Cao M.L., Zhang H.X., Zhang C. (2016). Effect of graphene on mechanical properties of cement mortars. J. Cent. South Univ..

[17] Zhang J.P., Liu L.M., Li Q.H., Peng W., Zhang F.T., Cao J.Z., Wang H. (2019). Development of cement- based self- stress composite grouting material for reinforcing rock mass and engineering application. Constr. Build. Mater..

[18] Liu X.S., Ning J.G., Tan Y.L., Gu Q.H. (2016). Damage constitutive model based on energy dissipation for intact rock subjected to cyclic loading. Int. J. Rock Mech. Min..

[19] Jin W.C., Chloé A. (2017). Discrete equivalent wing crack based damage model for brittle solids. J. Solid. Struct..

[20] Chen J., Ren S., Yang C.H., Jiang D.Y., Li L. (2013). Self-Healing Characteristics of Damaged Rock Salt under Different Healing Conditions. Materials.

[21] Miljkovi M., Radenberg M. (2016). Effect of compaction energy on physical and mechanical performance of bitumen emulsion mortar. Mater. Struct..

[22] Liu H., Yuan X. (2015). A damage constitutive model for rock mass with persistent joints considering joint shear strength. Can. Geotech. J..

[23] Guo W.Y., Tan Y.L., Yu F.H., Zhao T.B., Hu S.C., Huang D.M., Qin Z.W. (2018). Mechanical behavior of rock-coal-rock specimens with different coal thicknesses. Geomech. Eng..

[24] Gurvich M.R., Dibenedetto A.T., Ranade S.V. (1997). A new statistical distribution for characterizing the random strength of brittle materials. J. Mater. Sci..

[25] Wang X., Wen Z.J., Jiang Y.J., Huang H. (2018). Experimental study on mechanical and acoustic emission characteristics of rock-like material under non-uniformly distributed loads. Rock Mech. Rock Eng..

[26] Wang J., Ning J.G., Qiu P.Q., Yang S., Shang H.F. (2019). Microseismic monitoring and its precursory parameter of hard roof collapse in longwall faces: A case study. Geomech. Eng..

[27] Yang S.Q., Xu P., Ranjith P.G. (2015). Damage model of coal under creep and triaxial compression. Int. J. Rock Mech. Min..

[28] (2011). GB/T 1346-2011, Testing method for water requirement of normal consistency, setting time and soundness of the Portland cements. State Standard of the People’s Republic of China.

[29] (1999). GB/T 17671-1999, Method of testing cements-Determination of strength. State Standard of the People’s Republic of China.

[30] (2009). JGJ/T 70-2009, Standards for testing methods of basic performance of building mortar. State Standard of the People’s Republic of China.

[31] (2002). GB/T 50081-2002, Standard test methods of the mechanical properties of ordinary concrete. State Standard of the People’s Republic of China.

[32] Wang J., Ning J.G., Jiang J.Q., Bu T.T. (2018). Structural characteristics of strata overlying of a fully mechanized longwall face: A case study. J. S. Afr. I. Min. Metall..

[33] Solecki R., Conant R.J. (2003). Advanced Mechanics of Materials.

[34] Jin F.N., Jiang M.R., Gao X.L. (2004). Defining damage variable based on energy dissipation. Chin. J. Rock Mech. Eng..

[35] Gu Q.H., Ning J.G., Tan Y.L., Liu X.S., Ma Q., Xu Q. (2018). Damage constitutive model of brittle rock considering the compaction of crack. Geomech. Eng..

[36] Liu X.S., Tan Y.L., Ning J.G., Lv Y.W., Gu Q.H. (2018). Mechanical properties and damage constitutive model of coal in coal-rock combined body. Int. J. Rock Mech. Min..

[37] Yin Y.C., Zhao T.B., Zhang Y.B., Tan Y.L., Qiu Y., Taheri A., Jing Y. (2019). An Innovative Method for Placement of Gangue Backfilling Material in Steep Underground Coal Mines. Minerals.

[38] Jebli M., Jamin F., Malachanne E., Garcia-Diaz E., El Youssoufi M.S. (2018). Experimental characterization of mechanical properties of the cement-aggregate interface in concrete. Constr. Build. Mater..

[39] Gu Q.H., Ma Q., Tan Y.L., Jia Z.C., Zhao Z.H., Huang D.M. (2019). Acoustic emission characteristics and damage model of cement mortar under uniaxial compression. Constr. Build. Mater..

